# Ultra-Sensitive Automated Profiling of EpCAM Expression on Tumor-Derived Extracellular Vesicles

**DOI:** 10.3389/fgene.2019.01273

**Published:** 2019-12-17

**Authors:** Pouya Amrollahi, Meryl Rodrigues, Christopher J. Lyon, Ajay Goel, Haiyong Han, Tony Y. Hu

**Affiliations:** ^1^ Virginia G. Piper Biodesign Center for Personalized Diagnostics, The Biodesign Institute, Arizona State University, Tempe, AZ, United States; ^2^ School of Biological and Health Systems Engineering, Arizona State University, Tempe, AZ, United States; ^3^ Baylor Research Institute and Charles A. Sammons Cancer Center, Baylor University Medical Center, Dallas, TX, United States; ^4^ Molecular Medicine Division, The Translational Genomics Research Institute, Phoenix, AZ, United States

**Keywords:** liquid biopsy, extracellular vesicles, exosome, biomarker profiling, EpCAM, automated microscopy

## Abstract

Extracellular vesicles (EVs) are abundant in most biological fluids and considered promising biomarker candidates, but the development of EV biomarker assays is hindered, in part, by their requirement for prior EV purification and the lack of standardized and reproducible EV isolation methods. We now describe a far-field nanoplasmon-enhanced scattering (FF-nPES) assay for the isolation-free characterization of EVs present in small volumes of serum (< 5 µl). In this approach, EVs are captured with a cancer-selective antibody, hybridized with gold nanorods conjugated with an antibody to the EV surface protein CD9, and quantified by their ability to scatter light when analyzed using a fully automated dark-field microscope system. Our results indicate that FF-nPES performs similarly to EV ELISA, when analyzing EV surface expression of epithelial cell adhesion molecule (EpCAM), which has clinical significant as a cancer biomarker. Proof-of-concept FF-nPES data indicate that it can directly analyze EV EpCAM expression from serum samples to distinguish early stage pancreatic ductal adenocarcinoma patients from healthy subjects, detect the development of early stage tumors in a mouse model of spontaneous pancreatic cancer, and monitor tumor growth in patient derived xenograft mouse models of pancreatic cancer. FF-nPES thus appears to exhibit strong potential for the direct analysis of EV membrane biomarkers for disease diagnosis and treatment monitoring.

## Introduction

Tumor biopsies remain the gold standard for diagnosis, but are limited by how easily a tumor can be resolved and how amenable it is to biopsy. Surgical biopsies can also be costly, and incur risk and psychological stress. Analysis of tumor-derived or tumor-associated factors (e.g. circulating tumor cells (CTCs) and DNA (ctDNA), extracellular vesicles (EVs), and various protein biomarkers) in plasma or serum thus offer an attractive alternative. Such approaches have attracted substantial interest over the past decade for their ability to evaluate cancerous lesions, including primary and metastatic tumors, and permit serial analysis of their response to treatment.

CTCs and ctDNA are present in very low concentrations in blood, and their analysis require time-consuming and labor-intensive isolation techniques that are susceptible to contamination and lack specificity and batch-to-batch consistency (Yadavalli et al., 2017; Merker et al., 2018). CTCs cannot be detected in ~50% of cancer patients (Crawford et al., 2014) and CTC assays have been flagged for high levels of false-positive results (Alix-Panabières and Pantel, 2014). ctDNA assays have high specificity and moderate sensitivity for oncogenic variants (Mok et al., 2015; Hao et al., 2017), but may not reflect tumor heterogeneity and fail to detect scarce DNA variants (Bettegowda et al., 2014). In contrast to CTCs, ctDNA, and many proteins biomarkers, tumor-derived EVs are relatively abundant and stable in the circulation, contain factors that reflect the phenotype of their parent cell, and are secreted by all tumor cells and thus should reflect tumor heterogeneity (Kalluri, 2016).

The development of EV biomarker assays for cancer has, however, been limited by available assay methods. EV ELISA, a standard means of EV biomarker analysis, requires separate EV isolation steps, and its results are compromised by the lack of standardized and reproducible methods of EV isolation. Most EV isolation techniques subject EVs to forces or conditions that can alter their integrity, have poor and variable EV yields, and are subject to substantial variation in the purity and quality of their EV isolates. These issues are a major obstacle for the clinical translation of EV ELISAs, and their relatively large serum or plasma volumes requirements render them impractical for research involving mouse models, particularly studies that require serial blood draws.

Epithelial cell adhesion molecule (EpCAM, also known as CD326) represents a good candidate for an EV biomarker as it is overexpressed in many human adenocarcinomas and squamous cell carcinomas (Went et al., 2004), and this expression closely correlates with the epithelial-mesenchymal transition (EMT) regulating tumor invasion and metastasis (Gao et al., 2015; Ye et al., 2015; Sankpal et al., 2017). EpCAM is thus an appealing candidate biomarker for cancer diagnosis and the evaluation of anti-tumor therapy responses. Studies have employed different approaches to evaluate EV-associated EpCAM expression, including magnetic activated cell sorting (MACS) and microfluidic separation and analysis procedures (Runz et al., 2007; Rupp et al., 2011; Im et al., 2014; Zhao et al., 2016). However, all these the methods employed in these studies require ultracentrifugation for EV isolation, or large sample volumes, or employ complicated fabrication procedures and sensing methods. Development of a streamlined approach that uses common laboratory equipment to analyze EV biomarkers present in complex biological samples, therefore represents a critical unmet need for the development of EV biomarkers assays with potential clinical applications.

**Figure 1:**
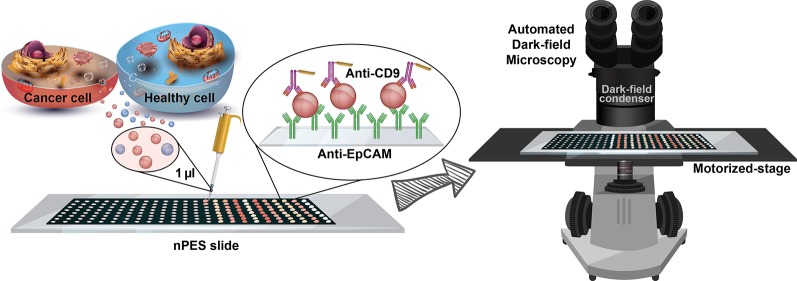
**–** Schematic of the isolation-free profiling of EpCAM-expressing EVs in small (1 µl) serum samples using a far-field dark-field (FF-DF) microscope assay, in which both image capture and analysis are automated to reduce assay variability and potential operator bias.

In this article, we describe a sensitive method that allows EpCAM expressing EVs to be directly analyzed from biological samples using a fully automated far-field dark-field (FF-DF) microscope to detect and quantify nanoplasmon-enhanced scattering (nPES) from nanoparticle probes bound to target EV biomarkers (**Figure 1**). Results of our study indicate that FF-nPES performance is similar to that of EV ELISA but does not require an EV isolation step. Proof-of-concept data indicate this assay can directly analyze EVs from microliter scale serum samples to detect the development of spontaneous pancreatic cancer and progression of human tumor xenografts in mouse models of pancreatic cancer and can distinguish patients with early stage pancreatic cancer from their healthy controls.

## Materials and Methods

### Cell Lines and Their Culture Condition

The human pancreatic cancer cell line PANC-1 was obtained from the American Type Culture Collection (Manassas, Virginia). PANC-1 cells were cultured in Dulbecco’s Modified Eagle’s Medium (DMEM; Hyclone, GE Healthcare Life Sciences) supplemented with 10% fetal bovine serum (FBS; Life technology, Thermo Scientific Inc.), 1 U·ml^−1^ penicillin, and 1 μg·ml^−1^ streptomycin (Corning). All PANC-1 cell cultures were grown in triplicate and incubated at 37°C in a humidified 5% CO_2_ incubator.

### EV Isolation From Cultured Media

PANC-1 cells were cultured to > 90% confluence, washed twice with phosphate-buffered saline (PBS; pH 7.0) and then incubated for 48 h in serum-free DMEM. Conditioned media was then collected and centrifuged at 2,000*g* for 30 min, supernatants were vacuum filtered using a 10 kDa centrifugal filter (Merck Millipore Ltd), and EV concentrates were centrifuged at 21,000*g* for 45 min to remove cell debris. Clarified supernatants were then centrifuged at 100,000*g* for 3 h and the resulting EV pellets were suspended in PBS (pH 7.0) and stored at 4°C and used within 48 h.

### EV Characterization

EV sample size distributions were measured using a NanoSight NS300 (Malvern Panalytical) equipped with a 532 nm laser, where EV samples were analyzed for 30 s, in triplicate, using NanoSight particle tracking software (screen gain and detection threshold set as 1.0 and 2, respectively). The morphology of EV samples negatively stained with osmium tetroxide were analyzed using a 2010F transmission electron microscope (JEOL USA). Western blots analyses were performed according to standard protocols using 20 µg (~ 8 μl) of cell and EV protein lysates that were probed with antibodies to TSG101 (4A10; Santa Cruz Biotechnology), VDAC1 (B-6; Santa Cruz Biotechnology), and EpCAM (VU-1D; Invitrogen), and a horseradish peroxidase (HRP)-coupled secondary antibody (RMG07; Abcam). Chemiluminescent signal from these Western blots was visualized using an ImageQuant™ LAS 4000 imaging system (GE Healthcare Life Sciences).

### Enzyme-Linked Immunosorbent Assay (ELISA)

Half-volume 96 well plates (3690; Corning) were incubated with 50 μl per well of the VU-1D9 anti-human EpCAM antibody (0.5 μg·ml^−1^ in PBS; Invitrogen) for 12 h at 4°C, then washed with PBS and blocked with 5% bovine serum albumin (BSA) in PBS supplemented with 0.01% Tween^®^ 20 (PBST, pH 7.0; Sigma-Aldrich) for 2 h. These plates were then aspirated and incubated for 12 h at 4°C with EV samples diluted in PBS (25 μl per well) to concentrations of 1.2, 0.8, 0.5, 0.4, and 0.2 µg/µl. Plates were then washed with PBST and incubated for 1 h at 37°C with 50 μl per well of biotinylated MEM-61 anti-human CD9 antibody (0.5 μg.ml^−1^; Invitrogen) suspended in 5% BSA/PBST. Sample wells were then washed five times with PBST and incubated for 1 h at 37°C with 50 μl streptavidin conjugated horseradish peroxidase (HRP, 1:5000 dilution, Cell Signaling Technology) suspended in 5% BSA/PBST. Plates were then washed five times with PBST and incubated for 15 min at 37°C with 50 μl per well of 3,3’,5,5’-Tetramethylbenzidine reagent (eBioscience Inc.), then supplemented with 50 μl per well of 2 M H_2_SO_4_ stop solution and analyzed for absorbance at 450 nm. The EV ELISA standard curve was calculated using GraphPad Prism 8.0.2 software (GraphPad Software) plotting optical density versus EV concentration.

### FF-nPES Assay of Serum Samples and Isolated EVs

192-well masked (8 rows x 24 columns) glass slides with protein A/G surface functionalization (nPES slides) were purchased from Arrayit Corporation. Each column represents 8 technical replicates of the same sample. Each well was filled with 1 µl of 0.5 μg.ml^−1^ EpCAM antibody; VU-1D9 anti-human EpCAM (Invitrogen) for isolated EV, and unpurified human and patient-derived pancreatic cancer xenograft (PDX) mouse serum samples and Clone G8.8R anti-mouse EpCAM (R&D Systems) for unpurified serum samples from KrasLSL.G12D/+; p53R172H/+; PdxCretg/+ (KPC) mouse models of spontaneous pancreatic cancer. Sample wells were incubated for 1 h at 37°C and washed three times with 1 μl per well of PBS. Slides were then blocked by addition of 1 μl per well of SuperBlock™ (Thermo Scientific™), incubated for 2 h at 37°C, and incubated for 4 h at 37°C with 1 μl per well of samples. Isolated EV samples were diluted in PBS to concentrations of 1.2, 0.8, 0.5, 0.4, and 0.2 µg/µl, with replicate samples added to each column (N = 8); while the human, PDX, and KPC serum samples were diluted with PBS at a 1:1 ratio. All wells were then aspirated and filled with 1 μl per well of PBS containing neutravidin functionalized gold nanorods (25 × 71 nm; Nanopartz™) conjugated with 0.5 μg.ml^−1^ biotinylated CD9 antibody, MEM-61 anti-human CD9 (Invitrogen) for isolated EV and for unpurified human and PDX serum samples and MZ3 anti-mouse CD9 (BioLegend) for KPC serum samples. To make these detection antibody probes, 40 μl of gold nanorods (~ 2.5 × 10^11^ particles) was washed with PBS three times (centrifuged at 8,500 g for 10 min), supernatant carefully removed, mixed with 10 μl of biotinylated antibody, and diluted to 200 μl with PBS. The mixture was stored at 4°C until use. After 1 h incubation at 37°C, slides were aspirated and washed once for 10 min each with PBST and de-ionized water on a HulaMixer (ThermoFisher Scientific), as described previously (Wan et al., 2019). The standard curve of the assay using isolated EVs was calculated using GraphPad Prism 8.0.2 (GraphPad Software) by plotting optical signal versus EV concentration. The EV EpCAM expression from human, PDX, and KPC serum samples was analyzed by plotting optical signal from replicate wells in each column of the nPES slide. Since samples in the first and last columns and first and last wells of each column of the nPES slides tended to exhibit edge effects due to greater evaporation effects in these wells, the first and last columns were filled with PBS and not used for analysis, and signals from the top and bottom row of each column were excluded from data analysis.

### Clinical Samples

Serum samples from pancreatic ductal adenocarcinoma patients and healthy donors were collected at the time of diagnosis by the biospecimen repository core lab at Baylor University Medical Center (Dallas, Texas) as specified by a protocol approved by its Institutional Review Board. All patients gave written informed consent for study participation. Only patients diagnosed with pancreatic ductal adenocarcinoma (PDAC) that underwent surgery (early stages) were selected for the sample collection and enrolled in this cohort. Investigators were blinded to the group identities of clinical samples during analysis.

Serum samples were rapidly thawed to room temperature from −80°C storage, vigorously mixed and then centrifuged at 500*g* for 15 min to precipitate protein aggregates and other debris. Supernatants were then diluted 1:1 with PBS, and vortexed before transferring 1 μl to each replicate well on the assay slide.

### Mouse Pancreatic Cancer Models

Serial blood samples were drawn every two weeks, for a total of four time points, from an 80-day-old male KrasLSL.G12D/+; p53R172H/+; PdxCretg/+ (KPC) mouse and three 4-month-old mice implanted with patient-derived pancreatic cancer xenografts (PDX mice) (Banerjee et al., 2016) housed at the Translational Genomics Research Institute in Phoenix, Arizona. Mice were analyzed to assess tumor development and size at each blood draw by three-dimensional high-resolution abdominal ultrasonography using a Visualsonics Vevo 770 system (Fujifilm Visualsonics, Ontario, Canada) as described (Whatcott et al., 2017). All animal procedures used in this study followed Institutional Animal Care and Use Committee (IACUC)-approved protocols and guidelines.

### Far-Field Dark-Field (FF-DF) Imaging and Optical Intensity Measurements

FF-DF images were captured using an Eclipse Ti-S inverted microscope (Nikon Instruments Inc.) equipped with a motorized stage, a 4× objective lens (NA = 0.13), and a dark-field condenser (1.20 < NA < 1.43) illuminated by a 100 W halogen lamp. FF-DF images of nanoparticle-scattered light were automatically captured using a DS-Ri2 color camera (Nikon Instruments Inc.) and automatically processed on Image J image analysis software (NIH) using the Dark Scatter Master (DSM) plugin (Sun et al., 2016) to avoid operator bias.

### Finite-Difference Time-Domain (FDTD) Simulations

FDTD simulations were performed using Lumerical FDTD Solutions software. The material optical properties of gold were set to the Johnson and Christy standard (Johnson and Christy, 1972). One unit-cell was simulated with a normal incidence of a plane wave source(s); while the in-plane and out-of-plane boundary conditions were set to periodic and perfectly matched layers (PMLs), respectively. Circularly-polarized light was simulated by a superposition of two linearly polarized sources with π/2 relative phase retardance. The mesh accuracy was set to 5 and a refined mesh was used around the gold nanorod. The auto shutoff for convergence of simulations was set to 10^−6^.

### Statistical Analysis

The data was statistically analyzed using one-way ANOVA, two-tailed Mann–Whitney U-test, and Student’s t-tests (significance level of α = 0.05) included in the GraphPad Prism 8.0.2 software.

## Results

### EpCAM Expression on EVs Isolated From a Human Pancreatic Cancer Cell Line

EVs were isolated from the pancreatic ductal carcinoma cell line PANC-1 to analyze the performance of this assay approach. Nanoparticle tracking analysis demonstrated that PANC-1 EVs isolated by ultracentrifugation had a mean diameter of ~130 nm and primarily overlapped the size range attributed to exosomes (30 – 150 nm) (**Supplementary Figure 1A**), while transmission electron microscopy found that these particles demonstrated the cup-shaped morphology characteristic of exosomes (**Supplementary Figure 1B**) (György et al., 2011). Western blot analysis found that EV lysates contained EpCAM and the cytosolic phosphoprotein tumor susceptibility gene 101 (TSG101) but not the mitochondrial membrane protein voltage-dependent anion-selective channel 1 (VDAC1), used as negative control for exosome samples (**Supplementary Figure 1C**) (Lötvall et al., 2014).

**Figure 2:**
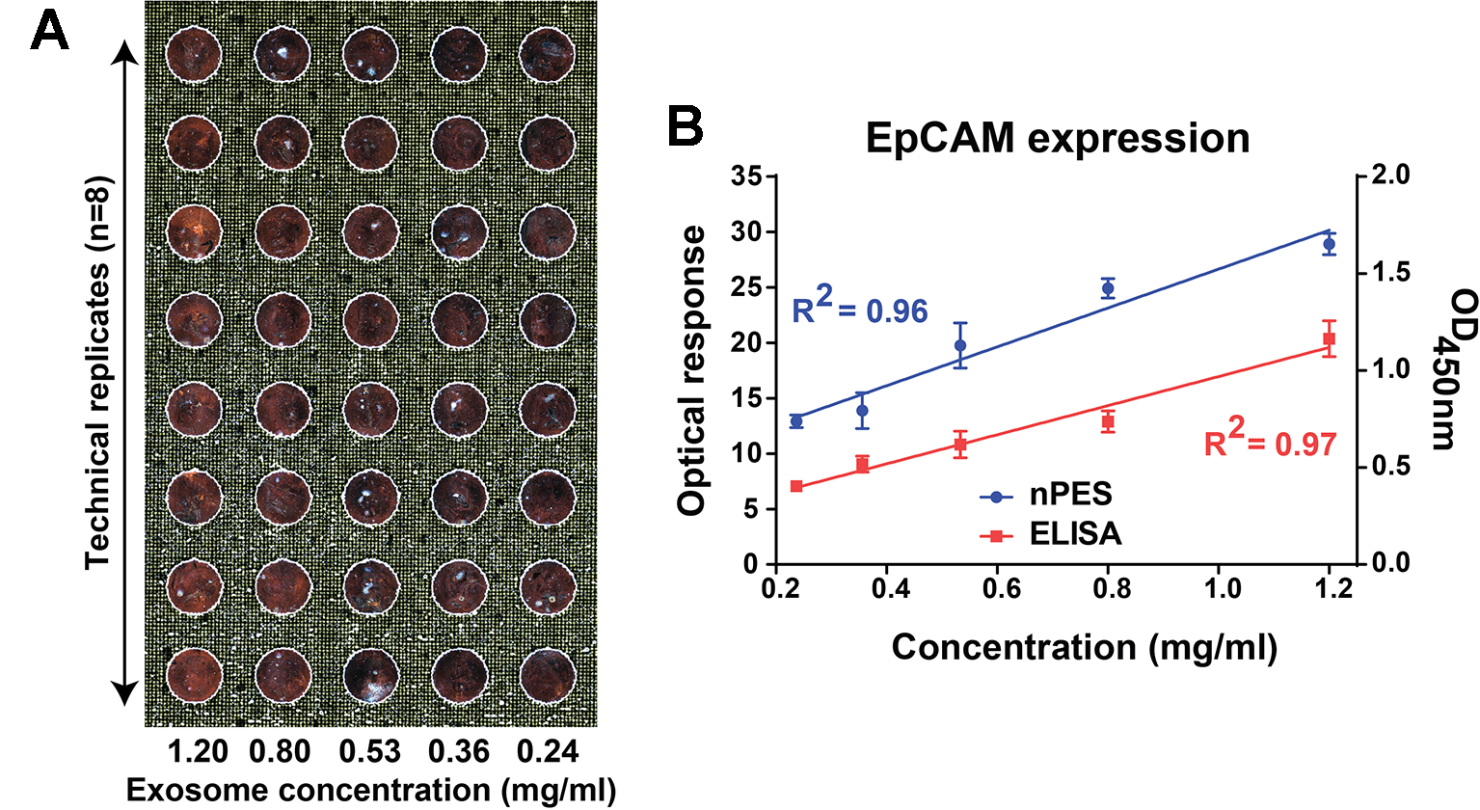
FF-DF image **(A)** and standard curve **(B)** of PANC-1 EV dilutions analyzed with an EpCAM capture antibody and an anti-CD9 specific AuNR probe. Data were analyzed as described in the *Materials and Methods* section and represent mean ± SEM; n = 6 replicates/sample.

Serial dilutions of PANC-1 EVs (1200, 800, 530, 360, and 240 ng·µl^−1^; approximately corresponding to 2.1, 1.5, 1.0, 0.6, and 0.3 × 10^9^ particles/µl, respectively) were immobilized on nPES slide by an anti-EpCAM capture antibody, hybridized with neutravidin-modified AuNRs that were pre-conjugated with a biotinylated anti-CD9 antibody, and target EV signal was quantified from far-field dark-field (FF-DF) images of these slides (**Figure 2A**). DF-FF optical signal revealed a strong linear correlation (R^2^ = 0.96) with EV concentration (mg/ml EV lysate) similar to that observed with an EV ELISA performed with the same samples, which required 5-fold more antibody (**Figure 2B**).

### EV EpCAM Expression in Two Mouse Models of Pancreatic Cancer

**Figure 3:**
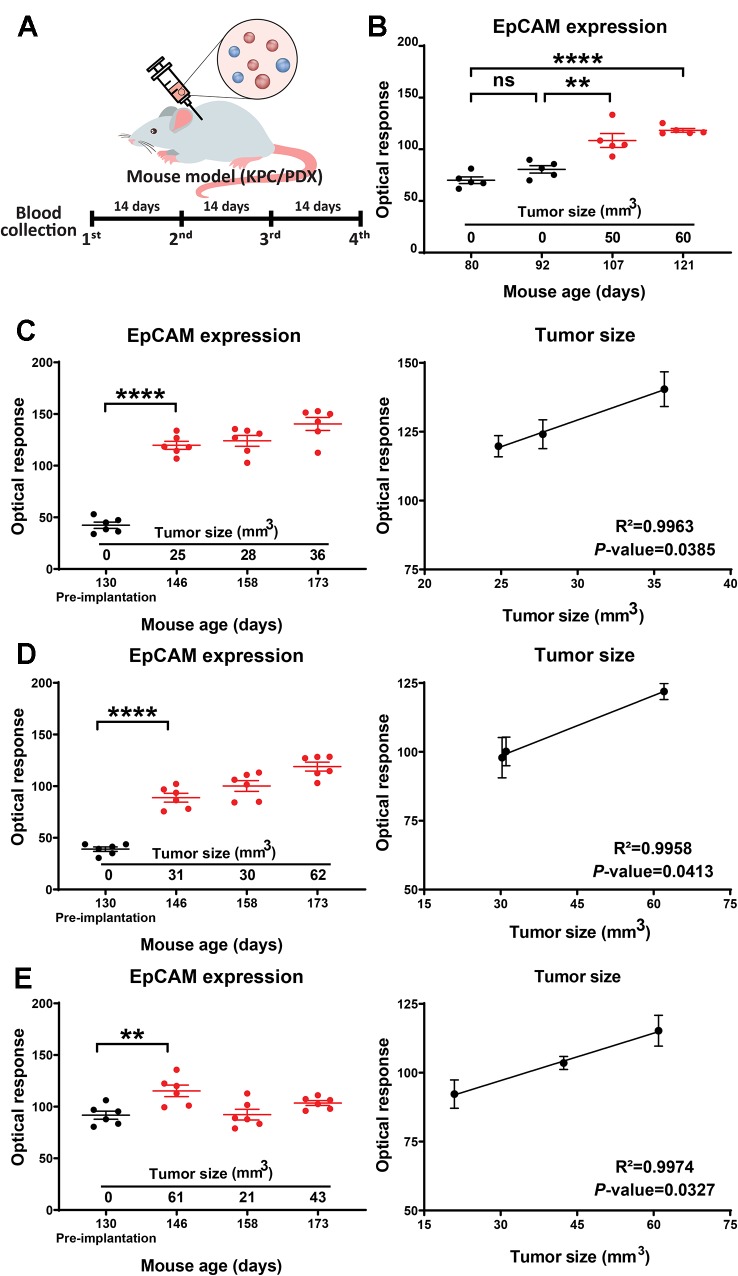
**(A)** Study timeline for the KPC and PDX mouse models. **(B)** EV EpCAM expression in longitudinal serum samples from a KPC mouse during tumor development. **(C**–**E)** EV EpCAM expression and their correlation with tumor size in longitudinal serum samples from PDX mice before and after tumor implantation. (Data were analyzed as described in the *Materials and Methods* section and represent mean ± SEM; n = 6 replicates/sample). **P-value = 0.0013, ***P-value < 0.0001; ns, P-value = 0.3331.

To evaluate the ability of our assay to detect EV EpCAM changes associated with tumor development or growth, KPC and PDX mouse models of pancreatic cancer were analyzed by serial blood draws and abdominal ultrasound procedures at four bi-weekly time points (**Figure 3A**). High resolution ultrasound analyses employed in this study were used to detect developing tumors and estimate their volumes. Both KPC and PDX mice were analyzed in this study, since KPC mice spontaneously develop pancreatic tumors (Hingorani et al., 2005) and represent a model of early tumor development, while PDX mice carry xenografts of human pancreatic tumors and thus directly reflect the genomic changes associated with human pancreatic cancer. EV EpCAM levels in the blood samples of the KPC mouse were found to significantly increase (*p*-value = 0.0013) at the first time point at which a pancreatic tumor mass was detectable (**Figure 3B**). EV EpCAM signal did not further increase at the subsequent time point, perhaps due to the modest increase in tumor volume in this interval. Blood samples were collected from three 130-day-old PDX mice before their tumor xenograft procedure and every two weeks thereafter. EV EpCAM signal was observed to significantly increase (*p*-value <0.005 for all mice) by two weeks after transplant and subsequent EV EpCAM signal was observed to correlate (R^2^ > 0.99 for all mice) with changes in the xenograft tumor volume (**Figures 3C**–**E**). Notably, this correlation held even in one PDX mouse that demonstrated tumor regression and subsequent expansion (**Figure 3E**).

### EV EpCAM Expression in a Cohort of PDAC Patients and Healthy Control Subjects

To investigate the performance of this FF-nPES platform to differentiate serum samples drawn from patients with and without pancreatic ductal adenocarcinoma (PDAC) based on their EV EpCAM signal, we analyzed serum samples obtained from a retrospective case-control study, where the case and control groups did not differ by age and gender (**Supplementary Table 1**).

**Figure 4:**
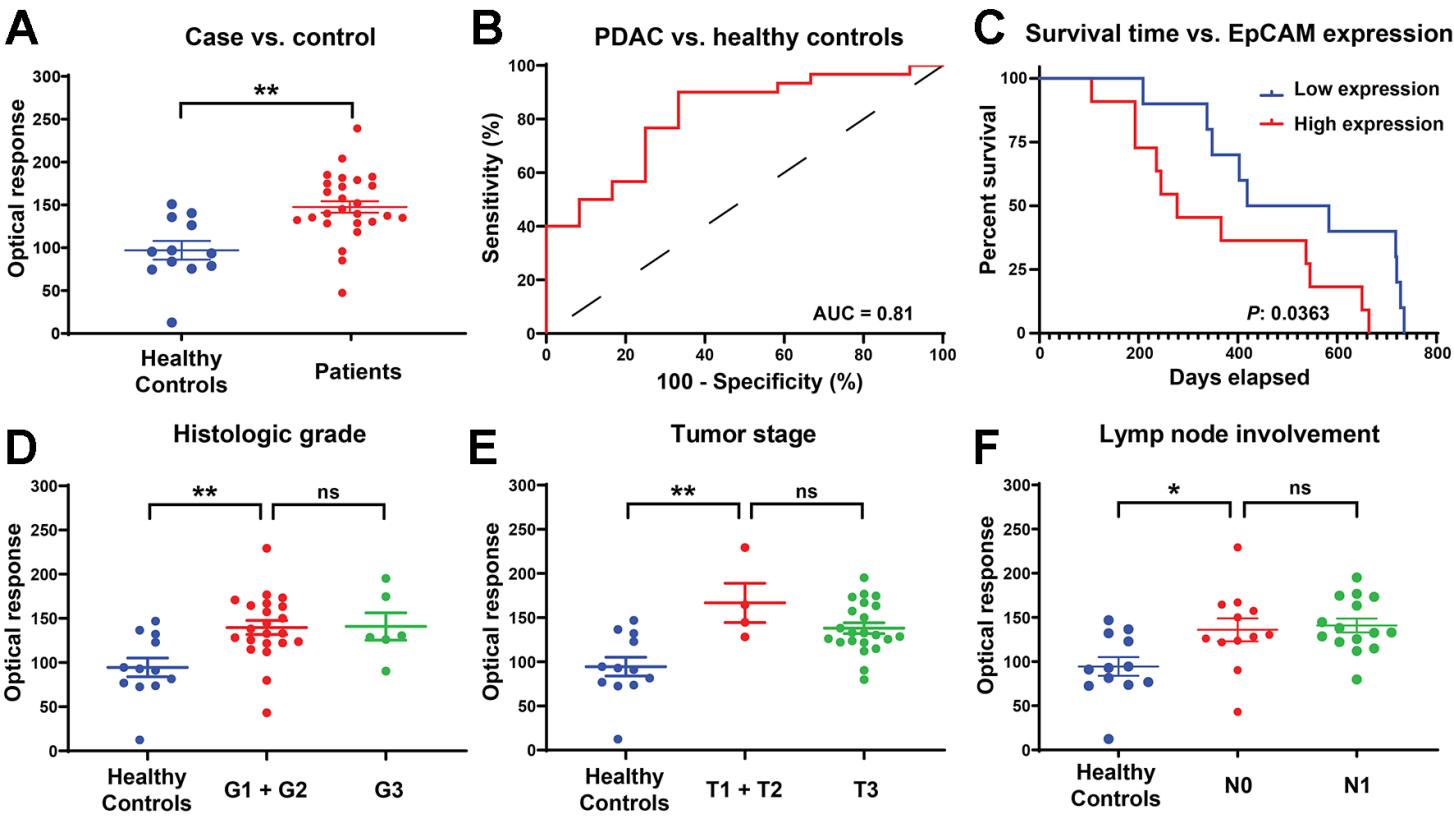
**(A)** EV EpCAM expression in serum samples from PDAC patients and healthy donors .**, P-value = 0.0011. **(B)** ROC curve of the ability to distinguish PDAC and healthy controls based on EV EpCAM signal. **(C)** Kaplan-Meier curve of overall survival time of patients with above average (high expression) and below average (low expression) serum EV EpCAM signal. **(D**–**F)** Difference in EV EpCAM signal in healthy controls and PDAC patients stratified by their histologic grade, tumor stage, and lymph node involvement (Data were analyzed as described in the *Materials and Methods* section and represent mean ± SEM; n = 6 replicates/sample). **(D)** **, P-value = 0.0053; ns, P-value = 0.9998. **(E)** **, P-value = 0.0018; ns, P-value = 0.3182. **(F)** *, P-value = 0.0299; ns, P-value = 0.9808.

FF-DF AuNR signal was significantly higher (*p*-value = 0.0011) in patients with PDAC versus healthy control subjects (**Figure 4A**), and a receiver operating characteristic (ROC) curve analysis (**Figure 4B**) found that this signal could effectively distinguish samples obtained from PDAC cases and healthy control subjects with good performance (area under the curve of 0.8139). Kaplan-Meier analysis of the PDAC cases after stratifying this cohort by high vs. low FF-DF EV EpCAM signal found that patients with high EV EpCAM signal had significantly reduced mean survival (520 vs. 365 days, *p*-value = 0.0363) (**Figure 4C**), despite failing to exhibit any apparent differences in their demographic or clinical information (**Supplementary Table 2**). Subsequent analysis found that EV EpCAM signal differed between healthy controls and PDAC patients with any histologic grade, tumor stage or degree of lymph node involvement, but was not significantly different among different levels of any of these tumor parameters (**Figures 4D**–**F**). EpCAM EV signal thus could not differentiate patients based on cancer stage but could distinguish patients with early stage PDAC from healthy controls. Pancreatic tumors are classified by multiple parameters, including histologic grading of its cellular differentiation state, its growth stage as assessed by their size and growth outside the pancreas, and its metastasis to nearby lymph nodes. FF-nPES results for serum EV EpCAM expression distinguished patients classified as having well-differentiated (G1) and moderately differentiated (G2) PDAC tumors, which exhibit slower growth and better prognosis that less differentiated tumors, from healthy controls but not from patients with poorly differentiated (G3) tumors (**Figure 4D**). FF-nPES analysis of serum EV EpCAM also distinguished patients with tumors smaller than 4 cm (T1 and T2) from healthy controls (**Figure 4E**), but not from patients with larger tumors. Finally, FF-nPES results also differentiated patients without lymph node tumor involvement (N0) from healthy subjects (**Figure 4F**), but not from patients with lymph node metastases (N1). Lymph node involvement is a strong predictor of poor outcomes, as median survival is markedly reduced (from 25.5 to 12.3 months) in pancreatic cancer patients with tumors that have metastasized to at least 2 lymph nodes (Baldwin et al., 2016). These results indicate that FF-nPES analysis of serum EV EpCAM levels can distinguish healthy subjects from patients with early stage pancreatic cancer by any of the three parameters analyzed in this study. This information did not resolve these early pancreatic cancer cases from more advanced cases defined by these same criteria, in agreement with the demographic analysis of patients with high and low serum EV EpCAM expression (**Supplementary Table 2**). It is thus unclear what is responsible for the reduced survival time observed between these groups, as differences in tumor severity do not appear to be responsible for this disparity.

## Discussion

Conventional tumor biopsy procedures can provide information about the genotype, biomarker expression profile, and differentiation state of the biopsy sample, but are useful only for accessible tumors and often carry risks that not all patients may be willing to accept. The information these biopsies provide is influenced by the portion of the sample that is analyzed, and such biopsies may therefore fail to capture the diversity of the tumor. Liquid biopsies, by contrast, utilize samples of body fluids (e.g. blood, cerebrospinal fluid, urine, saliva) that can be obtained by non-invasive or minimally invasive procedures that carry minimal risk. Such biopsies can be used to survey the cancer-associated factors released from tumors present in all anatomical regions, including surgically inaccessible sites. EVs represent a rich source of information in many liquid biopsy samples, including plasma and serum, since they are abundantly released by most tumors and are relatively stable in the circulation, unlike CTCs and ctDNA, which are present at low concentration and are relatively unstable in the circulation (Neumann et al., 2018). However, analysis of EV-associated biomarkers in liquid biopsies is complicated by the requirement that EVs be purified from these samples prior to their analysis, since there is no standard method for EV isolation, and most such methods are subject to substantial variations in EV yield and purity. FF-nPES circumvents this isolation step and employs a plasmon-based approach to detect exosomes captured by tumor-specific markers. This approach is modular, in that its specificity can be readily altered by changing the specificity of the capture antibody used to isolate the target EV population for analysis and employs a streamlined workflow that could be fully automated to reduce operator error and bias and enhance its potential for clinical translation. FF-nPES offers advantages over near-field nPES (Liang et al., 2017), which required an operator to identify and manually focus images used for analysis, increasing operator involvement and introducing potential bias. However, to offset this advantage, FF-nPES is less sensitive than near-field nPES, reducing its utility for the analysis of factors present in low concentrations.

Gold nanoparticles can be more readily functionalized than nanoparticles synthesized from other noble metals, without altering their size-dependent properties to scatter light at specific surface plasmon resonance (SPR) wavelengths (Polito et al., 2015; Wang et al., 2018). Gold nanorods (AuNRs) are more sensitive to their microenvironment than gold nanospheres (Rajeshwari et al., 2016) and are widely used as sensing elements (Vigderman et al., 2012; Wang et al., 2016). To improve FF-nPES sensitivity, we performed FDTD simulations to determine how the size of the AuNRs should be adjusted to maximize the nPES signal they produced at a given concentration (**Supplementary Figure 2A**). Nanoparticle SPR is governed by their absorption and scattering of light, and both properties are directly proportional to particle volume. Larger AuNRs scatter more light and are therefore more suitable for imaging applications (Park et al., 2014), although the detection efficiency of their SPR is also governed by the absorbance of their environment. Our FDTD simulation results indicated that the maximum SPR of AuNRs ~70 nm in length was not absorbed by water, indicating that these AuNRs had the best optical properties for detection probes in our FF-nPES EV assay platform. A 70-nm neutravidin-functionalized AuNR was therefore chosen as a probe to detect targeted EV surface proteins in a subsequent EV biomarker assay. In this approach (**Supplementary Figure 2B**), target EVs were captured by an antibody to EV biomarker of interest (i.e. EpCAM) that was immobilized to the surface of an assay slide, and captured EVs were visualized by an AuNR conjugated with an antibody that recognized a general EV surface marker (i.e. CD9).

FF-nPES appeared to demonstrate similar performance to EV ELISA, but offered several advantages not shared with EV ELISA, including the ability to directly analyze EVs from small volumes of serum. FF-nPES requires only a small volume of serum or plasma (<5 µL) to perform an EV analysis with eight technical replicates, much less than required by EV ELISAs. This low volume requirement makes FF-nPES a promising platform for the analysis of serum or plasma EV samples from mouse models, particularly in longitudinal studies that cannot employ EV ELISAs. FF-nPES assay results indicate that FF-nPES analysis of EV EpCAM levels in serial mouse serum samples can both detect the development of early pancreatic tumors and evaluate their progression. FF-nPES detected a serum EV EpCAM concentration change corresponding to the initial development of a spontaneous pancreatic tumor. This change was detected when the developing tumor was still 25 mm^3^, which matched the limit of detection of the high-resolution ultrasound approach used to detect and quantify tumors in this model. EV EpCAM concentration changes detected by FF-nPES also correlated with tumor growth in a PDX mouse model of human pancreatic cancer, and this correlation held even in one mouse that demonstrated tumor regression and regrowth, suggesting that this approach could have utility for the evaluation of tumor responses to therapy in drug or biomarker studies and potential clinical applications.

Both applications would be highly useful for the early diagnosis and monitoring of cancers, such as pancreatic cancer, that can be difficult to diagnose due to their nonspecific symptoms or which may be difficult to image or directly biopsy. Pancreatic cancer has one of the worst cancer survival rates, since its tumors are usually detected at later stages after they have metastasized to other tissues and are no longer curable by surgical resection (Howlader et al., 2012). Early diagnosis of this cancer is difficult, since its symptoms are shared by several other gastrointestinal tract pathologies. Imaging approaches used for tumor detection also fail to detect lesions < 3 cm in their largest dimension, and cannot be relied on for early detection (Kaur et al., 2012). Carbohydrate antigen 19-9 (CA19-9) and carcinoembryonic antigen (CEA) are the two most widely used markers for early diagnosis of PC, although only CA19-9 has received FDA-approval for pancreatic tumor evaluation. However, CA19-9 is not expressed by all patients with pancreatic tumors (only 65% demonstrate elevated CA19-9 levels while their tumors are still resectable) and patients with generic pancreatic diseases or other cancers can also exhibit elevated CA19-9 levels (Goggins, 2005). Therefore, other markers are gravely needed in the clinic for early stage diagnosis of pancreatic cancer. FF-nPES analysis of serum EV EpCAM expression may have potential for the early diagnosis and monitoring of pancreatic tumors if results from clinical trials match our mouse model results. Other studies have reported that changes in plasma EV EpCAM levels correlate with ovarian cancer (Im et al., 2014; Zhao et al., 2016) and breast cancer (Rupp et al., 2011).

FF-nPES offers several advantages over EV ELISA approaches in current use as a standard EV analysis approach in research studies, since it reduces assay time, variability, and the amount of sample volume required for an assay by eliminating the need for a separate EV isolation step. These features and its streamlined and automated assay protocol, which requires equipment that should be available in most well-equipped clinical laboratories make it a good potential candidate for clinical translation. Our proof-of-principle results suggest that such an assay could provide clinically useful data, but further analytical and clinical validation studies are required to determine its potential for adoption as an analysis platform in diagnostic EV biomarker applications.

## Data Availability Statement

All datasets generated for this study are included in the article/**Supplementary Material**.

## Ethics Statement

The studies involving human participants were reviewed and approved by Institutional Review Board at Baylor University Medical Center in Dallas, Texas. The patients/participants provided their written informed consent to participate in this study. The animal study was reviewed and approved by Translational Genomics Research Institute in Phoenix, Arizona.

## Author Contributions

PA and TH designed the study and experiments. PA performed the experiments, analyzed the data, and prepared the figures. MR performed the ELISA experiment. CL provided advice on data analysis. AG collected the clinical samples and formed the human cohort. HH collected the samples and tumor size measurement data from mouse models. TH supervised the study and provided advice on the research strategy and manuscript writing. PA and CL wrote the manuscript.

## Funding

The work was supported by research funding provided by NIH grants U01CA214254-01 set aside award, R01 AI122932 and R01 AI141500.

## Conflict of Interest

The authors declare that the research was conducted in the absence of any commercial or financial relationships that could be construed as a potential conflict of interest.
