# Supplementary figures and images for: Ultra-Sensitive Automated Profiling of EpCAM Expression on Tumor-Derived Extracellular Vesicles
Source: Front Genet. 2019 Dec 17;10:1273. doi: 10.3389/fgene.2019.01273 (PMC6928048; doi:10.3389/fgene.2019.01273)

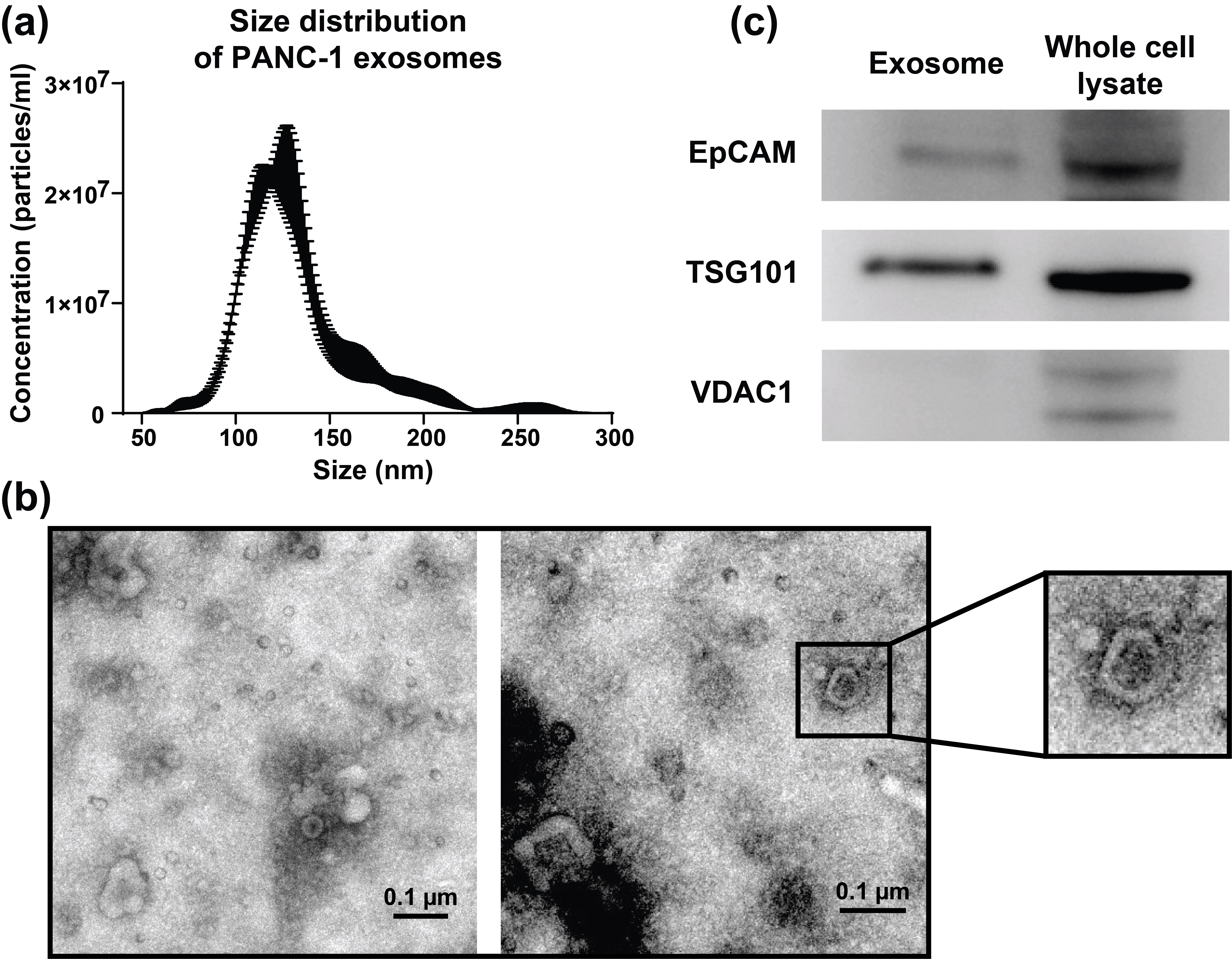

Supplement: Supplementary file 1 [file Image_1.jpeg]

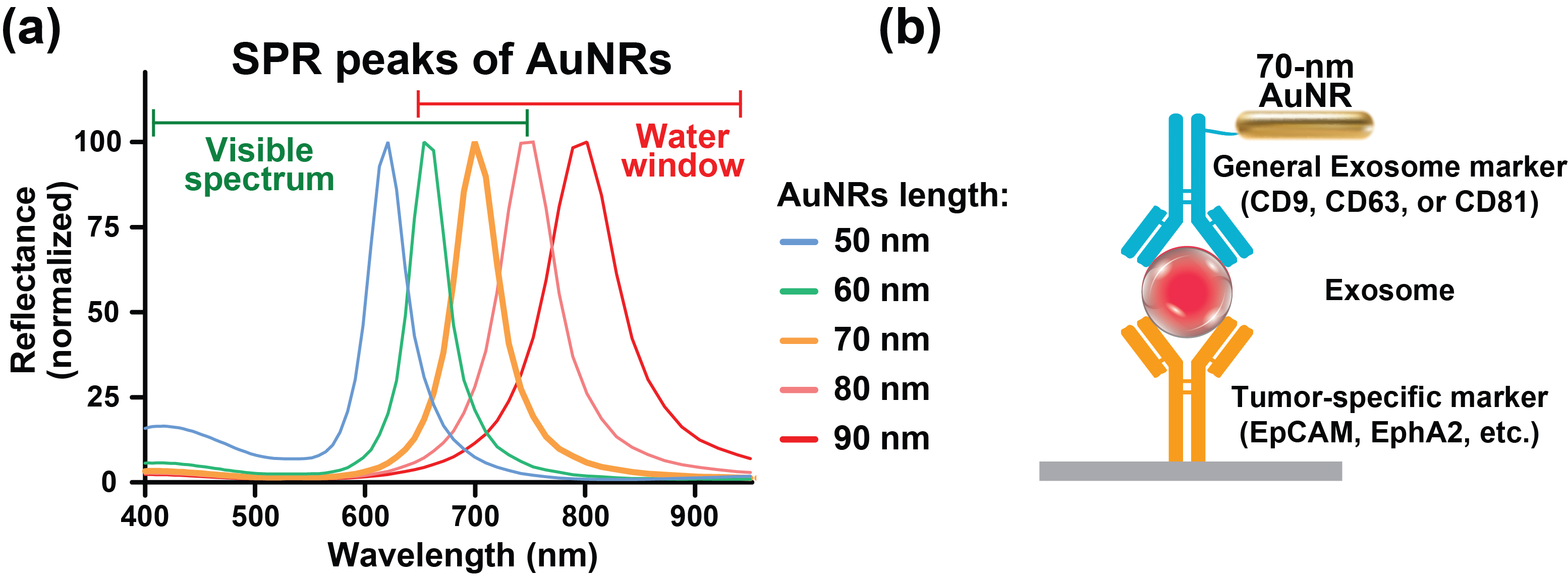

Supplement: Supplementary file 2 [file Image_2.jpeg]
