# Supplementary material for: Ultra-Sensitive Automated Profiling of EpCAM Expression on Tumor-Derived Extracellular Vesicles
Source: Front Genet. 2019 Dec 17;10:1273. doi: 10.3389/fgene.2019.01273 (PMC6928048; doi:10.3389/fgene.2019.01273)
Supplement: Supplementary file 3 [file Table_1.docx]

**Supplementary Table 1**

| Demographic characteristics of study participants. | | | | |
| --- | --- | --- | --- | --- |
| **Variables** |  | **Patient (n=30) n (%)** | **Control (n=12) n (%)** | ***P*-value^†^** |
| Age (years) |  | 66.3 ± 12.1 | 56.2 ± 15.7 | 0.0643 |
| Gender | Female  Male | 13 (43.3)  17 (56.7) | 7 (58.3)  5 (41.7) | 0.4994 |

† *P*-values are calculated by two-tailed Mann–Whitney U test for age and χ^2^ test for gender.
