# Supplementary material for: Ultra-Sensitive Automated Profiling of EpCAM Expression on Tumor-Derived Extracellular Vesicles
Source: Front Genet. 2019 Dec 17;10:1273. doi: 10.3389/fgene.2019.01273 (PMC6928048; doi:10.3389/fgene.2019.01273)
Supplement: Supplementary file 4 [file Table_2.docx]

**Supplementary Table 2**

| Demographic and clinical characteristics of study subjects stratified by EV EpCAM signal. | | | | |
| --- | --- | --- | --- | --- |
| **Variables** |  | **Low EpCAM expression** | **High EpCAM expression** | ***P*-value^‡^** |
| Age (years) |  | 59.4 ± 8.9 | 65.5 ± 13.2 | 0.1465 |
| Gender | Female  Male | 3 (37.5)  7 (53.8) | 5 (62.5)  6 (46.2) | 0.6594 |
| Tobacco use | Yes  No | 3 (60.0)  7 (43.8) | 2 (40.0)  9 (56.3) | 0.6351 |
| Diabetes | Yes  No | 1 (25.0)  9 (52.9) | 3 (75.0)  8 (47.1) | 0.5865 |
| Histologic grade | < G3  ≥ G3 | 6 (42.9)  4 (66.7) | 8 (57.1)  2 (33.3) | 0.6285 |
| Pathologic T stage | < T3  ≥ T3 | 1 (33.3)  9 (56.3) | 2 (66.7)  7 (43.8) | 0.5820 |
| Pathologic N stage | N0  N1 | 6 (66.7)  4 (40.0) | 3 (33.3)  6 (60.0) | 0.3698 |

^‡^ *P*-values are calculated by two-tailed Mann–Whitney U test for age and χ^2^ test for the rest of the variables.
